# Rats do not eat alone in public: Food-deprived rats socialize rather than competing for baits

**DOI:** 10.1371/journal.pone.0173302

**Published:** 2017-03-09

**Authors:** Omri Weiss, Alex Dorfman, Tamar Ram, Pazit Zadicario, David Eilam

**Affiliations:** Department of Zoology, Tel-Aviv University, Ramat Aviv, Israel; University of Lethbridge, CANADA

## Abstract

Limited resources result in competition among social animals. Nevertheless, social animals also have innate preferences for cooperative behavior. In the present study, 12 dyads of food-deprived rats were tested in four successive trials, and then re-tested as eight triads of food-deprived rats that were unfamiliar to each other. We found that the food-deprived dyads or triads of rats did not compete for the food available to them at regular spatially-marked locations that they had previously learnt. Rather, these rats traveled together to collect the baits. One rat, or two rats in some triads, lead (ran ahead) to collect most of the baits, but "leaders" differed across trials so that, on average, each rat ultimately collected similar amounts of baits. Regardless of which rat collected the baits, the rats traveled together with no substantial difference among them in terms of their total activity. We suggest that rats, which are a social species that has been found to display reciprocity, have evolved to travel and forage together and to share limited resources. Consequently, they displayed a sort of 'peace economy' that on average resulted in equal access to the baits across trials. For social animals, this type of dynamics is more relaxed, tolerant, and effective in the management of conflicts. Rather than competing for the limited available food, the food-deprived rats socialized and coexisted peacefully.

## Introduction

"**Let there be bread for all**"–Nelson Mandela

Competing for limited resources is a major driving force in the animal kingdom. In the context of social species, there is an apparent conflict between competing over resources on the one hand, and preserving group cohesion on the other hand. Indeed, living in groups has both benefits and costs, and a prerequisite for social species is to establish a balance between cooperation and competition among individuals. Group living usually involves the establishment of various social ranks and, accordingly, the distribution of resources is biased toward the highly-ranked individuals. Even then, however, conflicts among group members arise, with the two main conflicts being over mating partners [1] and food [2]. In the context of the latter, reducing competition over food resources is crucial for survival in social species. For example, reducing resource competition is vital for colonial seabirds in order to ensure self- and chick-provisioning during the breeding season [3]. Rats (*Rattus sp*.), including laboratory rats, are social animals in which dominance and subordination traits have been described [4–7]. Nevertheless, rats were shown to display various types of reciprocity [8–12] and even empathy [13–16]. Indeed, the notion of competing over limited access to food resources as a tool to assess dominance was criticized, and it was suggested that winning access to the limited resources does not necessarily represent dominance or high social rank, but merely reflects a better performance of some individuals over others [17–18], winners, however, are more likely to win subsequent conflicts [19]).

As noted above, recent studies have revealed that rats with limited access to food may display a type of prosocial behavior rather than provoking competition [8, 13–14, 16, 20], and even help unfamiliar rats (generalized reciprocity [9–10]; for a theoretical treatment of this mechanism see [21]). Those studies demonstrated that social factors may dominate the desire for preferred food in rats. Furthermore, it was argued that rats perform prosocial behavior toward both familiar and unfamiliar rats, and that such performance is repeated consistently and intentionally day after day and at shorter and shorter latencies [15]. It was also suggested that social animals have evolved strong innate preferences for cooperative behavior [22], and that a specific behavior is not a mere product of the proximal immediate cost and benefit, but it also has an ancestral component, balancing the gain in an ultimate evolutionary success in addition to the immediate gain [23]. In other words, prosocial behavior in rats is a reflection of a desire for social contact [24–25] (see however [16]).

A past study with rat dyads revealed that the spatial choices of individual rats may affect the future spatial choices of their partners in a foraging task [6]. In another study, in which dyads of food-deprived cage-mate rats were conflicted between competing for limited resources and retaining social contact with their partner, it was demonstrated that the rats unequivocally favored remaining with their partner rather than splitting up to forage independently [26]. Interestingly, while one of the rats ran ahead and ate most of the pieces of the food, the other rat systematically traveled with the leader rather than splitting up from the dyad and foraging independently ("spatial segregation"; [3]). Altogether, past studies have revealed that rats first and foremost favored to travel together even when they were expected to compete over limited resources and, accordingly, it is unlikely that their behavior reflects competition.

In the present study we expanded the previous studies [8, 13–14, 16, 20] by taking 12 dyads of cage-mate food-deprived rats and testing them in four successive trials in which one rat in each dyad collected more baits. The same rats were then randomly divided into eight triads, each with three food-deprived rats that were unfamiliar to each other, and which had again to compete for limited equispaced pieces of food. Accordingly, we posed two questions: (i) would the "leaders" in the first dyad trial preserve their "leadership" in subsequent dyad trials and the triad trial; and (ii) would the triad of food-deprived unfamiliar rats split up and compete for the food, or travel and forage together as they had done in a previous study [26]? Answering the first question was expected to reveal whether "leadership" in foraging rats is a personal trait or merely a transient better performance; while answering the second question might uncover whether socializing or competing depends on familiarity with the other individuals.

## Materials and methods

### Animals

Twenty-four male Sprague–Dawley rats (age 6–7 months; weight 450–600 g) were housed in a temperature-controlled room (22 ± 1°C) under an inverse 12/12-h light/dark cycle (dark phase 8:00–20:00). Rats were held in standard rodent cages (40 x 25 x 20 cm; two rats per cage) with sawdust bedding and were provided with *ad-libitum* access to water and standard rodent chow. Under these housing conditions rats were exposed to the odors and sounds of all other rats, but had visual and tactile contact only with their cagemate. For each cage, rats were marked with a waterproof marker on their tail, one rat with a single stripe and the other with a double stripe. Before testing they underwent daily handling for two weeks.

#### Ethics note

We confirm that this study was carried out in strict accordance with the recommendations of the Guide for the Care and Use of the Institutional Animal Care and Use Committee (IACUC) of Tel-Aviv University, Permit Number L-14-051. In this permit, Tel-Aviv IACUC approved the specific procedures in this study. No animals were sacrificed for the purpose of this study.

### Apparatus

**Fig 1:**
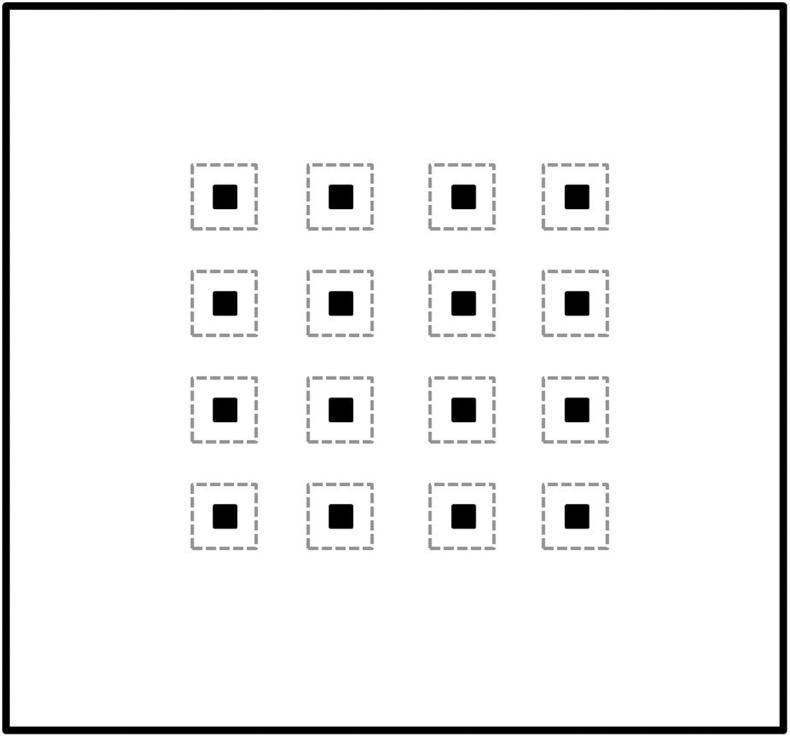
A plot of the arena, objects, and zones. The large circumference represents the 6 x 5.6 m arena, and the 16 dark squares (■) represent the 16 equispaced objects. The dashed square around each object represents the 36 X 36 cm object zone.

Rats were tested in a 6 x 5.6 m arena, comprising the white floor of a light-proofed air-conditioned room (22 ± 1°C). The room door had the same cover of that of the walls, and was located 50 cm above the floor so that there was no distinct visual or tactile landmark on the room perimeter. The room was illuminated with four cool-white LED projectors (65W each), sufficient to distinguish between subjects but subtle enough to prevent discomfort to the rats. Sixteen objects (each a 12 x 12 x 6 cm cement cube) were placed in a grid layout, equispaced at 90 cm from each other in the center of the arena (see Fig 1). Trials were recorded by four equispaced Mintron MTV-73S85H color CCTV cameras, placed 2.5 m above the arena, each providing a top view of one of the arena quarters. The four video images were integrated and tracked as one image by a tracking system (Ethovision XT 10; Noldus Information Technologies, NL) at a rate of five frames per second.

### Procedure

Training and testing were carried out during the dark phase of the rats’ dark/light cycle, in order to test the rats during the period when they are most active. Each rat underwent a series of training sessions preceded by 12 hrs of food deprivation with access only to water. Fifteen minutes before each session, rats were brought to a room adjacent to the apparatus and their backs were gently painted in blue, green, or red with a waterproof marker, enabling the tracking system to differentiate among them. Each of the 16 objects was then baited with a small piece of chocolate-flavored cereal, placed in the center of the top surface of each object. An individual rat was then placed gently in the near right corner of the arena, and the experimenter left the room. Dyads and triads of rats were each hand-held by one or two experimenters and gently released simultaneously near the right corner of the arena, with all of them facing the arena center. Training sessions continued until each rat had collected food from at least 14 objects in less than 20 min. Each rat underwent a different number of training sessions depending on its learning rate (mean ± SEM = 3.60 ± 0.15 training sessions). When three rats from different cages had completed the training sessions, they underwent two additional sets of 15-min trials: (i) **'dyad trials**', in which cage-mates were tested together four times in the course of two weeks, (two trials/week); (ii) a **‘triad trial’**, in which three unfamiliar rats (from different dyads) were tested together. Before the dyad set of trials and before the triad trial, each rat also underwent a 15 min '**lone trial**', which was used as a reference for its behavior in the social trials. In this procedure, each rat had learned the location of baits in the test arena before being tested in it with one familiar or two unfamiliar partners. At the end of each trial the rats were returned to their original cages and the arena was mopped with soap and water in order to neutralize odors prior to the next session.

### Data acquisition and analysis

Data acquisition was performed automatically for all rats, and the experimenter was blind to the role of the rat in previous trials. For the lone and dyad/triad trials the following parameters were extracted from ‘Ethovision’, and further analyzed with Microsoft Excel 2010 and STATISTICA 8 (Statsoft, UK):

*Cumulative distance*: The cumulative metric distance traveled during the 15 min trial.
*Task duration*: Time elapsed between the first arrival at the first object and the first arrival at the 16th object.
*Distance traveled during the task*: The cumulative metric distance traveled by a rat during task duration, which was usually shorter than the 15 min trial duration.
*Distance traveled along the walls*: The cumulative distance traveled within a 40 cm zone along the four arena walls (away from the object zones).
*Latency to arrival at the first object*: The time elapsed between the beginning of the trial and the first arrival at any one of the objects.
*Total visits to the objects* (repetitions included): The cumulative number of visits to objects in the course of the entire trial (arrival at the object zone was considered as a visit).
*Number of objects visited (repetitions excluded)*: The cumulative number of visits to different objects in the course of the entire trial (the possible range is from 0 to 16 objects).
*Number of collected baits*: Scrutiny of the video files revealed that the first rat to arrive at a baited object collected the bait. Only in three cases did a rat that was first to arrive at a baited object not collect the bait. Therefore, for each rat, the number of first arrivals at baited objects was considered as the number of collected baits.
*Visit duration*: The time (sec) lapsed from arrival at a zone until leaving that zone.
*Duration between objects*: The average duration between first visit to one object and first visit to the next, previously unvisited, object.
*Leader and Follower*: We used these terms literally to describe which rat ran ahead of the other(s) to collect more baits ("leader"), and which rat was trailing behind the "leaders" in collecting baits. As noted in the 'Discussion', leadership in one domain (collecting baits) rarely predicts leadership in other domains.

### Statistics

One way ANOVA was used to compare the behavior of the same rats across trials. Two-way ANOVA was used to compare the behavior of leader and follower rats (between-group effect) in the lone and triad trials (within-group effect). For this, rats in the lone trials which preceded the first dyad trial were classified as "leaders" and "followers" according to their behavior with partners in the first dyad trial.

## Results

### A comparison of group and individual performance

**Fig 2:**
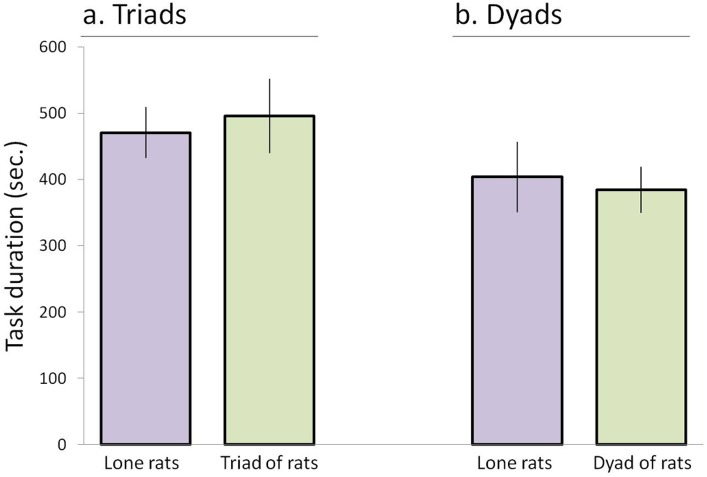
**The mean (± SEM) of task duration (the time to reach all 16 objects) is depicted for rats in triads and their preceding lone trial (a) and for the rats in the first dyad trial and their preceding lone trial (b).** Duration for lone rats refers to the task duration when rats were tested as individuals in the lone trial. Duration for triads refers to the arrival at all 16 objects by any of the rats, implying that each rat could hypothetically visit only some of the objects, as also applicable to the dyad data. As shown, there was no significant difference in task duration between lone rats and triads/dyads.

Rats in the dyad/triad trials could have divided the task among them, with each consuming a different set of baits, enabling them together to accomplish the task of consuming the 16 baits faster than the lone-trial rats. For example, each of the three rats in a triad could have collected baits from 5–6 objects and the triad could thereby complete together the task of collecting all the baits faster than lone individuals. However, this was not the case and the duration of consuming the 16 baits by two or three rats together (regardless of which rat collected them) did not differ from the performance of the same individuals in the lone preceding trials (Fig 2). Indeed, a one-way ANOVA comparison of task duration in the first dyad trial and the triad trial revealed no significant difference (F_3,64_ = 0.98; p = 0.4091). Task duration was thus shown not to differ whether one rat, a dyad of rats, or a triad of rats had to collect the 16 baits. Implicit in these results is that rats in dyads and triads did not split up to collect the baits independently, but traveled together, as illustrated in Videoclip 1 in which the rats are observed to be more preoccupied in socializing than in collecting the baits.

### Leading and following as episodic states that change over trials

Table 1 presents the number of baits collected by each individual rat across the four dyad trials and the subsequent triad trial. As shown, there were two rats that collected more than half of the baits in all trials (top two rows in Table 1). Another three rats consistently collected just a few baits (bottom three rows). The other 19 rats displayed substantial changes from one trial to the next in terms of collecting baits. Thus, leading or following in bait collection were only episodic transient phases for 19 out of the 24 rats, for which either leading ore following was preserved within a specific trial and changed in subsequent trials.

**Table 1:** The number of baits consumed by each of the 24 rats (rows) during the four dyad trials and the triad trial. As shown, two rats (top two rows) were continuously leading in terms of the number of baits they collected. Another three rats (bottom three rows) were followers, always collecting a few baits. The other 19 rats greatly varied in the number of baits they collected across trials.

Dyad trials				Triad trial	
1st	2nd	3rd	4th		
14	16	16	16	9	Always leaders
11	11	11	12	14	
14	9	8	10	1	Transient states of leading or following
13	14	9	2	0	
12	16	12	14	3	
12	13	9	2	11	
12	9	1	3	9	
11	6	9	9	0	
10	9	1	7	8	
10	5	0	3	7	
9	14	12	7	4	
9	7	6	14	0	
7	9	10	2	7	
7	2	4	9	7	
6	11	16	13	12	
6	7	15	9	1	
5	10	7	6	1	
4	7	15	13	9	
4	3	7	14	6	
2	7	8	6	3	
2	2	7	12	7	
5	5	5	4	5	Always Following
4	0	4	2	1	
2	0	0	0	3	

**Fig 3:**
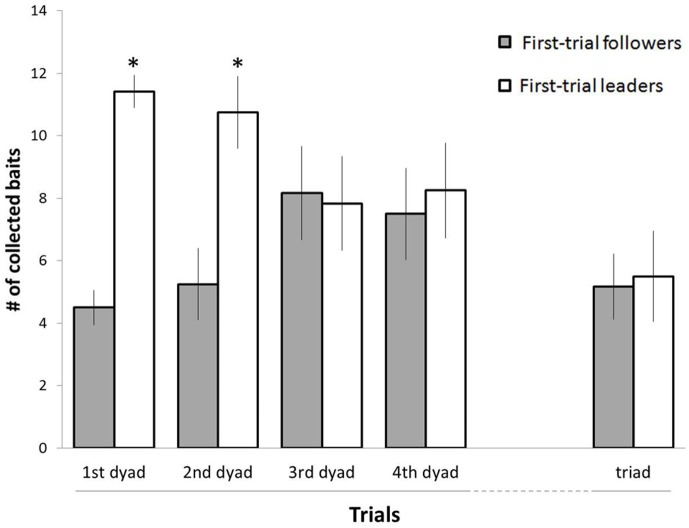
The mean number (± SEM) of baits collected by leaders and followers across trials. Rats were categorized as leaders or followers in the first dyad trial (left hand columns). These first-trial categories as leaders and followers were retained for the subsequent trials, regardless of the actual number of baits collected by the rats in these subsequent trials. The mean of the actual performance in subsequent trials is thus depicted according to the original categories, illustrating a diminishing difference between leaders and followers, reaching equity from the third trial on. * indicates a significant difference between the leaders and followers in that trial, as well as between the leaders in this trial and both leaders and followers in the triad trial. Note that this does not mean that there were no leaders and followers from Trial #3 on. Conversely, there were always leaders and followers but their identity changed.

Further, each rat was categorized as a "leader" or a "follower" according to the number of baits it had collected during the first dyad trial, with leaders collecting more than 8 baits and followers less than 8 baits. Retaining this assignment into "leaders" and "followers", we then calculated how many baits were collected by each of the 'first-trial leaders' and 'first-trial followers' in the subsequent trials. The means (± SEM) for these data, depicted in Fig 3, show that the difference between these groups, which was apparent in the first trial, diminished over successive trials and leveled off from the third dyad trial onwards. Indeed, a two-way ANOVA with repeated measures revealed a significant difference between leaders and followers (F_1,88_ = 6.57; p = 0.0276), a significant difference between trials (F_4,88_ = 2.79; p = 0.0312), and a significant interaction of trial X leader/follower states (F_4,88_ = 5.58; p = 0.0005). A Tukey HSD post-hoc test revealed that the number of baits collected by leaders in the first and second trials significantly differed from the number of baits collected by followers in these trials, as well as from the number of baits collected by either leaders of followers in the triad trial (Fig 3). Notably, as shown in Table 1, leading and following states were exchanged between most of the rats, so that a running-ahead rat in one trial typically became a follower in the next trial, and vice versa. The episodic states of forerunning and following were thus preserved within a trial but exchanged and leveled off across trials. Furthermore, we compared the changes between each two successive trials with 3,000 randomly-generated changes within the same range. A two-way ANOVA with repeated measures revealed a significant difference between the randomly-generated changes and the actual changes shown in Table 1 and Fig 3 (F_1,9066_ = 7.24; p = 0.0072), a significant difference between the changes between successive trials (F_3,9066_ = 29.08; p < 0.0001), and a significant interaction (F_3,9066_ = 3.88; p = 0.0088). Nevertheless, a HSD post-hoc test for unequal n revealed that the only change that significantly deviated from the random data was between the fourth dyad trial and the triad trial, since the average number of baits collected by a rat dropped since there were now three rather than two rats sharing the 16 baits. Nevertheless, the lack of significant difference between randomness and the changes among trials 1–4 attests for the inconsistency of the leader and follower states over repeated trial.

The question arose as to whether it was possible to identify "leaders" and "followers" already in the preceding lone trial, when they were tested individually before being exposed to the baits together with partners. For this, data of the rats during the triad trial were divided into leaders and followers in accordance with their performance in the triad trial, and compared for the lone and triad trials (Table 2). A repeated-measure two-way ANOVA was performed and followed by a Tukey post-hoc test. As shown, there was no significant interaction. Notably, there were a few within-trial significant differences (comparing leaders and followers). These parameters are depicted in the shaded rows of Table 2. Specifically, there were significant differences between leaders and followers in four parameters: leaders traveled a greater distance during the task of visiting the 16 objects, had a shorter latency to visit the first object, visited more different objects (repetitions excluded), and overall paid more visits to all objects (repetitions included). None of these differences, however, were reflected in the individual rats in the lone trial, implying that the "leadership" of these rats was manifested only in the presence of conspecifics. Similarly, the behavior of leaders also demonstrated a significant differences (in four out of the eight parameters in Table 2: 'Cumulative distance', 'Distance traveled during the task', 'Visit duration' and 'Distance traveled along the perimeter'), whereas the behavior of followers differed in only one parameter ('Cumulative distance') between the triad and lone trials. Overall, there was no significant interaction in the eight parameters depicted in Table 2, indicating that the trend of change in each parameter was similar in leaders and followers, but that these changes were more salient in leaders compared with followers.

**Table 2:** Mean (± SEM) data on eight activity parameters for rats in the lone and the subsequent triad trial. In each trial, rats were classified as leaders or followers according to their performance in the triad trial. For each parameter, the results of a two-way ANOVA are depicted at the right for within-trial comparison (between leaders and followers), for between trial comparison (between lone and triad trials), and for the interaction of trial x leading. Significance is depicted in boldface. The results of a post-hoc Tukey HSD comparison are depicted in superscript, as specified at the bottom of the Table.

Parameter	Lone trial		Triad trial		Within trial F_1,22_ P	Between trials F_1,22_ P	Interaction F_1,22_; P
	Leader	Follower	Leader	Follower			
**Cumulative distance (m.)**	118.4 ± 6.1	111.8 ± 4.6	145.1 ± 4.6^2^	129.7 ± 5.7^2^	1.3; 0.26	**31.53 < 0.001**	1.26; 0.27
**Distance traveled during the task (m.)**	65.5 ± 4.5	59.5 ± 3.4	97.9 ± 4.4^1^^,^^2^	70.6 ± 6.1	**7.11; 0.01**	**10.28; 0.003**	2.45; 0.13
**Latency to first object (sec.)**	49.6 ± 8.5	92.3 ± 14.8	93.9 ± 13.1	144.3 ± 14.2	**5.8; 0.023**	**8.6 0.007**	0.05; 0.810
**Task duration (sec.)**	363.8 ± 40.9	374.7 ± 23.8	521.1 ± 31.6	412.6 ± 36.4	1.5; 0.235	3.4; 0.076	1.2; 0.268
**# of visits to different objects (repetitions excluded)**	14.4 ± 0.8	12.2 ± 0.9	15.3 ± 0.2^1^	10.4 ± 0.9	**13.3; 0.001**	3.6; 0.068	3.6; 0.068
**Total # visits to the objects** **(repetitions included)**	29 ± 1.5	22 ± 1.43	31.93 ± 1.6^1^	20.1 ± 2.1	**12.53; 0.001**	0.07; 0.8	1.56; 0.22
**Visit duration (sec.)**	14.8 ± 1.4	10.1 ± 1.5	8.6 ± 0.6^2^	6.4 ± 0.6	3.53; 0.07	**11.7; 0.002**	0.68; 0.41
**Distance traveled along the perimeter (m.)**	45.9 ± 3	54.5 ± 3.2	62.9 ± 3.3^2^	68.2 ± 4	1.49; 0.23	**18; <0.001**	0.22; 0.65

^1^Significantly different from leader in the same trial; Tukey HSD test

^2^Significantly different from its behavior in the lone trial; Tukey HSD test

**Fig 4:**
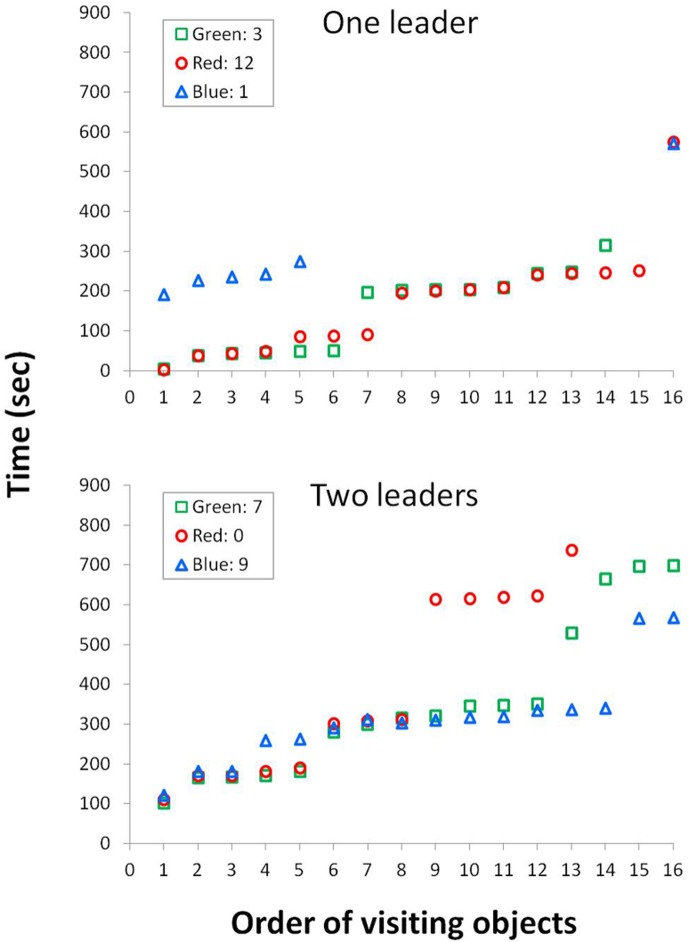
The tendency of the rats to travel together with one or two partners to the same objects is illustrated for a triad with one leader (top) and a triad with two leaders (bottom). The 16 objects are ranked on the abscissa according to the order in which each was visited, and the time of visiting each of the objects by each of the rats is given along the ordinate. Accordingly, for each object the order of symbols from bottom to top (time) reflects the order in which the rats arrived at that object. In the triad with one leader, the red rat (**○**) arrived first at objects 1–3, and then again at objects 7–15. The green rat (**□**) arrived first at objects 4,5,6, and the blue rat (**△**) arrived first only at object 16. Notably, the red and green rats arrived at most objects almost at the same time while the blue rat was out of the race most of the time. In the triad with two leaders (bottom), the green rat (**□**) arrived first at objects 1–7, and then the blue rat (**△**) took over and arrived first at objects 8–16, while the red rat (**○**) always lagged behind. As shown, the three rats traveled together to the first eight objects, arriving at them almost at the same time; then the green and the blue rats continued to travel together to objects 9–12 while the red rat split up from the triad; and, ultimately, the blue and the green rats also split up, with the blue rat arriving a few minutes before the green rat at objects 13–16. The data for triads with two leaders indicate alternating "leadership" with none having consistent precedence as in triads with one leader. Altogether, the overlap and adjacency of the symbols of the different rats (arrival time) illustrate their arrival at the same object at the same time.

Followers trailed the leaders; in 53% of the arrivals of a leader to a baited object, a follower rat arrived at the same object within 15 seconds, and in another 21% of arrivals the follower rat arrived at the same object in less than one minute. In 43% of arrivals of the leader to a baited object, the third rat also arrived at the same object (see for example Vidoeclip I and Fig 4). Notably, there was no difference in the total activity of leaders and followers (top row in Table 2). In other words, all the rats, leaders and followers, were similarly active (and as shown in Videoclip 1 and Fig 4 also traveled together), with the leaders collecting more baits and paying overall more visits to the various objects (whether baited or not). The tendency of the rats to arrive together with partner(s) at the same objects is illustrated for two triads in Fig 4. As shown, the leadership of one or two rats in the triad is conspicuous.

### Rats tended to travel with partner(s) to the objects

In most trips to the objects, leader and follower rats tended to travel with one or two partners (75% and 76% of all trips, respectively). Moreover, in each of the shared trips, leaders and followers visited together most of the objects visited in that trip (74% and 73%, respectively). Altogether, the rats took most of the trips to objects together, mostly visiting the same objects in each of these shared trips. Similarly, the baits were usually collected during shared trips (see videoclip 1). Only 26% and 37% of the baits were collected, respectively, by leading and following rats during trips without partners, when only one of the rats was in the object zone and the other rat (or the other two other rats in the triad trial) were in the perimeter zone. These data demonstrate the tendency of the rats to travel together and visit the same objects in the same trips, regardless of being a leader or a follower. In other words, the rats that collected more baits during the trial ("leaders") did not acquire their status by traveling alone, but mainly by traveling ahead of their partners in shared trips.

In triads in which two of the rats were first to arrive at about the same number of objects, these leading rats shared, on average, about 79% ± 5% of the objects in each trip. That is, they mostly traveled together, temporally exchanging leadership between them, with being first at some point and eventually collecting the same number of baits (Table 1 and Fig 4). Leading and following were thus episodic states in which rats traveled mostly together, traversed about the same overall distance, and underwent similar changes to their behavior compared with their behavior in the lone trial. Nevertheless, these changes were more salient in leaders, conferring upon them their episodic "leader" status in being first to access the baited objects during a specific trial. Altogether, rats with partners interacted with each other both before and in-between approaching the objects, whereas the same rats in the lone trials approached the objects immediately or after progressing along the arena wall (see Videoclip 1).

Videoclip 1.

## Discussion

In the present study, 24 food-deprived rats were first trained individually to collect baits placed on each of 16 equispaced objects. Having learned to collect the baits, they were then tested with the 16 baited objects first as cage-mate dyads over four trials, and afterwards as triads of three rats that were unfamiliar to one another. We found that when tested in dyads or triads, the rats did not split up to collect the baits independently, but mostly traveled together to the various objects, with either one, or two of them in some triads, leading and arriving first at the majority of objects and collecting the baits. Nevertheless, regardless of which arrived first, the rats mostly traveled together (Videoclip 1) with no substantial difference among them in terms of their total activity. It would seem that rats in dyads and triads focus more on socializing, tending to travel to the objects with partner(s). In consequence, the time taken to collect all 16 baits was approximately the same for lone rats, dyads, or triads of rats. In terms of collecting baits, leading and following states in individual rats were exchanged over repeated trials. In other words, leading and following in collecting the baits were transient states that were usually preserved within a trial [specific session of testing a dyad] but changed across trials (subsequent testing of the same dyad). In the following discussion we interpret the puzzling preference of the food-deprived rats to travel together rather than splitting up and collecting the baits independently. We suggest that the change in "leadership" over trials, as observed in the present study, may reflect a sort of 'peace economy' in which all individuals equally benefit from the available resources over trials.

The present results offer a follow-up to our previous studies, which showed that rats prefer to travel together [24] and that food-deprived rats favor socializing over competing for food [26]. As in the previous studies, here too it seems puzzling as to why the food-deprived rats in triads foraged together rather than competing for the food available to them at the regular spatially-marked locations that they had previously learned. It could be argued that the rats were not hungry, but this is unlikely since for such a relatively small mammal, 12-hr of food deprivation is not trivial. Another possibility is that the rats traveled together while competing for the baits, with some leading in one trial and others leading in subsequent trials, demonstrating a sort of "episodic personality" [27]. This, however, is less likely since the rats continued to travel together to the objects even after they had consumed all the baits and there was no apparent benefit for rats except that of social traveling. This indicates that there is a strong social affiliation among rats, and in the present experiment it overweighs hunger, which is a basic and strong drive in animal behavior, and the past knowledge on the location of palatable food. Altogether, foraging and collecting the baits was definitely not the rats' only motivation in traveling through the arena. Since over trials however, all get similar amount of baits, and since this was gained without apparent competition, we termed it "peace economy".

The present results do not provide substantial support for leadership and followership in rats. A social hierarchy with dominant and subordinate individuals characterizes rats, including laboratory rats [5, 28]. It was presumed that competition over limited access to food presents a measure of competitive dominance [29]. This notion was examined in further studies, revealing that in the case of limited resources, foraging performance does not necessarily reflect dominance/subordination ("*the fallacy of limited access to food and dominance*"[17–18]). Even the use of the terms 'leader' and 'follower' was criticized and replaced with 'high-performing rats' and 'low-performing rats' [30–31]. A recent survey on leadership in mammals has highlighted several dimensions of leadership [32], characterizing a spectrum of various types and intensities of leadership. Applying these criteria to the present study, it would seem that in the context of a specific trial, some rats may be considered as achievement-based moderate leaders that coordinate the behavior of the others (followers), with the payoff (baits) skewed to these "leaders". Leadership in one domain (collecting baits), however, rarely predicts leadership in other domains (at least in the other behavioral parameters that were measured in the lone and triad trials). Indeed, of the various activity parameters that were measured in the present study, none could predict which rat would be a leader or a follower. Therefore, even if collecting more baits in a specific trial could be considered as representing a specific type of leadership or an aspect of dominance, this better performance was only a transient state, with the rats changing between or among them this limited type of leadership and followership over trials.

When a rat dyad or triad was traveling, one or two of the rats followed and collected only a few baits, and sometimes not even one, yet the rats kept traveling together. A possible explanation for this could be that of the model of spontaneous emergence of leadership in foraging pairs [33]. According to this model, the rat with lowest reserves determines when the group should forage, while the other rats that follow are likely to benefit from the safety of a joint activity, along with the possibility that they too may forage. Accordingly, group coordination emerges spontaneously by means of temporary ‘leaders’ and ‘followers’, due to individual differences in the energetic states, with a simple rule of thumb: "I forage if either my reserves have fallen below a certain threshold value, or my partner chooses to forage" [33]. Moreover, this tactic for collective travel requires only the ability to observe and react to a change in the partner’s behavior–a type of self-organized behavior [34] that emerges spontaneously. Restricting the term "leadership" to literally moving ahead thus legitimates the simple rule of "follow the individual that moves first", and automatically produces leaders and followers [35–36]. This model may also explain why leadership and followership states were stable within a specific trial, but changed across trials, so that typically, a leader in one trial could become a follower in the subsequent trial, and vice versa. In the context of the latter model, it could be argued that the present results are due to scrambled competition in which one individual is faster in depleting a limited resource, but another individual then takes over since, for example, the first individual is now engaged in digestion (see appendix in [37]). This is not likely, however, since the rats could recover from hunger after each trial with *ad-libitum* access to food, and were again food-deprived only a few days later, before the subsequent trial.

Rats tend toward social affiliation, as revealed in early reports [4, 7], and in the present task they were also motivated to obtain food. It could be argued that when a rat traveled towards a baited location, it was sometimes followed by another rat that was motivated by the social affiliation tendency. Nevertheless, the changing states among or between the rats across trials in most rats may reflect prosocial behavior or a type of reciprocity, as recently revealed in both wild and laboratory rats [8–12, 20, 38]. Specifically, it was suggested previously that interactions in which followers voluntarily follow the leaders reflect leader-follower relations that are more reciprocal and mutually beneficial [35]. Moreover, it was also argued that the change in leading-following states over trials is in line with the mutually beneficial exchange between a cooperator and a reciprocating partner [39], which is a form of direct reciprocity–a basic form of reciprocal cooperation [[35]. Indeed, recent laboratory studies demonstrated that wild rats display a generalized reciprocity that does not depend on the identity of the recipient [8], a direct reciprocity in which the partners are familiar [9, 11, 14], and that they help the more hungry recipients or those in poor condition [10]. Other studies have demonstrated reciprocity in laboratory rats, suggesting that the mechanism for this reciprocity is a display of food-seeking behavior [40], and that prosocial behavior in rats occurs even in the absence of strategic, reciprocal, or selfish motivations [38]. It was also suggested that the origin of this behavior is more likely to be genetic than cultural [41]. Notably, the present results show a lack of competition, which could be a result of a sort of reciprocity although the latter is not explicit in the present findings. Nevertheless, we suggest that the past studies on reciprocity [8–12, 20, 38], together with the present results, may be viewed as a sort of "peace economy" by the rats, aimed at increasing the welfare of the individuals. The present results, however, demonstrate only that rats preferred to forage together and that they displayed balanced food consumption over trials. Notably, for small mammals such as rats, the primary pay-off in within-group cooperation may be the anti-predator benefit offered by 'safety in numbers' [42]. Rats may therefore cooperate even when they only benefit sometimes, since by working together they are less likely to become victim to predation. Accordingly, laboratory rats, which notably display reduced aggressiveness compared to their wild conspecifics, did not compete for the food in the present study despite being food-deprived. Rather, they displayed a sort of 'peace economy' that on average resulted in changing "leadership" among or between them and equal access to the baits across trials (Table 1 and Fig 3). Indeed, it was suggested that this type of social dynamics is more relaxed, tolerant, and effective in the management of conflicts. It is achieved through a process in which individuals continually modify social relationships in order to attain a peaceful coexistence [43].

## Supporting information

S1 Videoclip (WMV)

S1 File (XLSX)
